# Pathology and Morbid Anatomy

**Published:** 1900-12

**Authors:** 


Pathology and Morbid Anatomy. By T. Henry Green, M. D., F. R. C. P., Physician and Special Lecturer on Clinical Medicine at Charing Cross Hospital, etc. New (9th) American from 9th English edition. Revised and enlarged by H. Montague Murray, M. D., F. R. C. P., Lecturer on Pathology and Morbid Anatomy at Charing Cross Hospital. Revised for America by Walton Martin, Ph. B., M. D , of the College of Physicians and Surgeons, New York City. Octavo volume of 578 pages, with 4 colored plates and 339 engravings. Philadelphia and New York: Lea Brothers & Co. [Price, cloth, $3 25 net.]

For many years Green's pathology and morbid anatomy has stood for excellence and superiority in the field of pathological research, and no better evidence of this can be adduced than in the presentation of the ninth enlarged edition. In spite of the fact that since the first edition was published, many authors have entered this field, nevertheless, Green's has passed from one edition to another, each keeping pace with the advances taking place and each improving on its predecessor.

In preparing a new edition of this favorite text-book, nearly one-half of the subject matter has been rewitten and many new sections have been added by the English editor, Dr. H. Montague Murray. In order to adapt the work still more fully to the needs of American students, it has been carefully edited by Dr. Walton Martin, who has supplied complete chapters on malaria and on the blood and has added a chapter on the preparation and staining of tissues for microscopic study.

The effective series of illustrations has been increased by 180 engravings and some colored plates, so that taken as a whole this standard work has been virtually re-created and will be found to respond to the wants of the student to even a greater degree than did its eight preceding editions.

The book is nicely bound in moroon colored linen, well put together and printed on heavy paper. — W. C. K.

